# WHO recommendations for pregnancy and postnatal care clarified

**DOI:** 10.2471/BLT.26.296273

**Published:** 2026-10-08

**Authors:** Mercedes Bonet, Maria Barreix, Özge Tunçalp, Anayda Portela

**Affiliations:** a Department of Sexual, Reproductive, Maternal, Child, Adolescent Health and Ageing, World Health Organization, Avenue Appia 20, 1211 Geneva 27, Switzerland.; b Institute of Tropical Medicine, Antwerp, Belgium.

A recent article in the *Bulletin of the World Health Organization* highlighted the importance of hypertensive disorders of pregnancy as one of multiple conditions that affect pregnant and postpartum women and their babies.^1^ We appreciate the authors’ engagement with World Health Organization (WHO) guidance and their contribution to ongoing dialogue on how best to support countries in improving maternal and newborn health outcomes. We welcome the opportunity to clarify the scope and purpose of WHO’s recommendations for antenatal and postnatal care for a positive pregnancy and postnatal experience, and the associated adaptation toolkit.

WHO recommendations on antenatal and postnatal care define the routine care that all pregnant and postpartum women, as well as their babies, should receive. These recommendations are part of, and complementary to, a broader portfolio of guidelines covering essential care and the prevention, detection and management of leading maternal and newborn complications, including hypertensive disorders during pregnancy and postpartum. Under the guidance of an external technical advisory board, maternal and newborn health recommendations are regularly reviewed and prioritized for updating based on emerging evidence, implementation experience and country needs.^2^^–^^4^

In 2025, WHO published the antenatal and postnatal care adaptation toolkit^5^ to respond to country demand,^6^ based on the scope of recommendations for a positive pregnancy and postnatal experience. The toolkit provides standardized methods for country programmes to review and prioritize global recommendations and adapt them to national and local contexts. The toolkit is intended to complement the *WHO recommendations on antenatal care for a positive pregnancy experience*^7^ and *WHO recommendations on maternal and newborn care for a positive postnatal experience*^8^ on routine care and on the management of specific conditions, rather than to be used as a standalone tool. The toolkit was co-developed with policy-makers, health workers, implementers and managers of maternal and child health services, and was tested in diverse settings. The toolkit has now been used in 12 countries in the African Region and South-East Asia Region to adapt and develop national guidelines and care packages for routine antenatal and postnatal care, including the review of treatment and management of pregnancy and postnatal complications. During country adaptation processes, national authorities can link additional relevant condition-specific guidance according to local priorities to the adapted guidelines. Strengthening linkages across WHO recommendations and implementation tools is important to support integrated, quality maternal and newborn care. Further work is planned to strengthen the components on screening and clinical assessment for signs and symptoms of complications in routine care, and adequate prevention, management and follow-up based on condition-specific guidelines.

WHO is advancing work to further integrate up-to-date recommendations across the continuum of care, including but not limited to hypertension during pregnancy, pre-eclampsia and eclampsia. Some examples are the forthcoming update of the *WHO-UNICEF-UNFPA Integrated management of pregnancy, childbirth, postnatal and newborn care (IMPAC) – essential and emergency care: a clinical practice guide*,^9^ and the update of the antenatal and postnatal adaptation toolkit. The update of this practice guide will incorporate recommendations published since 2025 and those related to prevention and management of common complications, including noncommunicable diseases, within antenatal and postnatal care service platforms. In addition, any updated version of the toolkit will be reviewed with end-users and tested in different settings before its release. At global level, WHO and other partners have expressed their commitment to addressing hypertensive disorders of pregnancy, and recent efforts aim to ensure alignment across research, normative guidance, access to health products, implementation, advocacy and accountability.^10^^,^^11^
